# Editorial

**Published:** 1875-08

**Authors:** 


### Editorial.

### AMALGAM.

In the Transactions of the New York Odontological Society for December, 1874, are three articles upon the subject of amalgam, as a material for filling teeth. Each of these papers possesses some interest, since they make the impression, if they do not make the direct claim that they are based upon, and involve a great amount of experience, research and experiment. We now have only a few thoughts upon the first article, that by Dr. S. P. Cutler, of Memphis, Tenn. What he is aiming at in the paper, is not very clear. Is it to prove that the common amalgam or any amalgam constitutes a good filling for decayed teeth? If so, the course he takes is a very strange one for every experiment he gives or refers to, bears upon its face strong evidence against the material for this purpose. He says, "Filings of Platinum and mercury rubbed together in the palm of the hand do not amalgamate," * * * they do not form a metallic mass at all, but remain in the form of a dark powder." Is a black powder a good material with which to fill teeth? Again "I combined platinum with silver and tin in small proportion and found that just in proportion to the amount of platinum was the amalgamation retarded." * * "So long as there was any platinum present, so long was the process retarded."

Such an amalgam is certainly not good. Again, "The next experiment was with platinum and gold, one equivalent of platinum and two of gold; amalgamation was very slow, requiring considerable rubbing. After the lapse of two weeks, it was not firm, but was easily picked to pieces with the finger nail; in fact it did not harden to any extent, * * I considered this form useless in practice."

His next experiment is, "One-fourth part platinum and one-half part gold, ordinary amalgam, equal quantity to both. These amalgamated, the process being very slow and difficult. * * This was of grayish-white, rather dirty in appearance, and did not become hard and firm."

Certainly no one would claim that this is a good or even admissible material for filling teeth. In his fifth experiment he takes, "One equivalent of platinum, one equivalent of gold and four equivalents of common amalgam of equal parts of silver and tin * * ; twenty-four grains of this, combined with sixteen of mercury pressed in the hand only. This was much whiter than the other preparations, and amalgamated much sooner. * * There was a good deal of dark matter, no doubt from the tin."

"After twenty-four hours, weather temperate, the lump had lost one grain, was white and firm, but not hard enough for durable work; in twenty-four hours longer, the mass had become quite as firm as ordinary amalgam. This would answer for front teeth, badly decayed, as it would hold its color quite well, but be no better for back teeth than common amalgam."

Was it the "temperate weather" that removed the deal of dark matter and made the lump white and firm? Temperate weather is good on some things, but we did not know that it would knock the dark spots out of amalgam. If the amalgam will answer for badly decayed front teeth, why will it not answer just as well and better for front teeth not badly decayed? Or is extensive and bad decay, an element of success in the use of this material? It would seem so from the oft repeated statements that are made in this direction.

Or is this particular amalgam good for badly decayed front teeth because it has a good deal of dark matter in it, that would be positive and well defined and so more sightly than the sickly hue of a badly decayed front tooth? If this amalgam is good for front teeth why is it not good for molars and bicuspids?

The Doctor by his experiment rather condemns copper amalgam for filling teeth, he says: "Copper filings, rubbed with mercury, do not easily amalgamate, * * does not harden at all." That fixes that matter. Next!

"Brass filings, after considerable rubbing, amalgamate slowly, but quicker than copper, owing to the zinc in the brass. This specimen did not harden in the least." But it beat the copper. Brass amalgam is not reliable for filling teeth!

"Nickel does not unite with mercury after long rubbing, hence could be of no use in amalgams." This statement is quite satisfactory. However, would *short* rubbing help the matter any.

Again, "Cadmium combined with common amalgam, would only injure the preparation, from the fact of its readiness to oxidize in the mouth, forming a soluble oxide, which would endanger the pulp of the tooth." Another very objectionable compound.

In another paragraph he says: "I next tried Dr. E. A. Bogue's formula, as given in the April number of the Dental Advertiser. Twenty-four grs. of amalgam alloy and seven grs. of mercury, rubbed together in the hand, as usual, formed a gray powder, which would not form a mass by ordinary pressure, * * but by heating the instrument, consolidation was made perfect. * * After filling a cavity in this way and removing immediately, it fell to powder as before filling. Upper teeth could not be easily filled with this powder."

We will accept this as a correct conclusion.

Now these embrace a portion of the experiments made by Dr. Cutler as recorded in his paper before the Odontological Society at New York, and so far as they go, to prove anything, it is that amalgam is a very unsuitable material for filling teeth. The remaining experiments and his statements upon the subject, contained in the latter part of his article, we will reserve for another time.

### MICHIGAN UNIVERSITY DENTAL COLLEGE.

At the last session of the Michigan Legislature, an appropriation was made for the establishment of a Dental Department in the Michigan University, following which the Regents of the Institution established such a department and have appointed professors and teachers, and are proceeding to complete the arrangements as rapidly as possible; and it is proposed to have all things in readiness for operation by the 1st of Oct., next. The course will begin at and occupy the same time (viz, six months) as the medical term.

The following requirements are established: A course of of three years pupilage with two full courses of college instructions, three are advised. For admission a satisfactory examination, as to literary attainments. For graduation a good moral character; a satisfactory examination upon all the branches; taught together with good evidence of skill and ability for the speciality.

All the aids and facilities of the University are at the service of the student. The hearty co-operation of the dental profession of Michigan, with the University, in this enterprise, will result in the establishment of one of the best Dental Colleges in the world. Instruction upon the various branches will be given by the following Professors:

J. B. Angell, L. L. D., President.

Corydon L. Ford, M. A., M. D., Anatomy and Physiology.

Alonzo B. Palmer, M. A., M. D., Pathology.

Donald Maclean, M. D., Surgery.

F. H. Gerrish, M. A., M. D., Therapeutics and Materia Medica.

E. S. Dunster, M. A., M. D., Diseases of Women and Children.

Silas H. Douglas, M. A., M. D., Chemistry, and Director Chemical Laboratory.

J. Taft, D. D. S., Principles of Operative Dentistry.

J. A. Watling, D. D. S., Clinical and Mechanical Dentistry.

W. H. Jackson, Demonstrator.

### CELLULOID—A NEW APPARATUS.

The reports upon the value and efficiency of celluloid incrcase in strength and number daily. The improvements in the material and the apparatus for working it, and the growing knowledge of, and increased skill in working it, about warrant the assertion that it is one of the established resources of the profession.

The amount of the material sold is largely on the increase. One large dental depot reports that they sell celluloid, for quite as many dentures, as rubber, and the celluloid goes to a far larger number of dentists than the rubber. The facilities for working it are continually being increased. It is now worked more successfully in dry heat than in steam or oil. A dry heater invented and constructed by Dr. Talbot, of Chicago, is certainly a very effective apparatus. There is no possibility of an explosion, the progress of the work can be observed and any irregularities corrected. The plates come from the plaster in far better condition, so far as smoothness is concerned, when worked in dry heat. It is also found that there is no difficulty in uniting it. We recently saw a plate composed wholly of small fragments of the material, and it seemed about as perfect as any plate.

The warping, springing and drawing from the teeth have all disappeared under the present skill and knowledge. Celluloid is for more pleasant to work, and vastly better in the mouth We have not yet seen the mucous membrane of the mouth,. affected by contact with a celluloid plate.

### SENSITIVE DENTINE.

Use "Pain obtunder" for sensitive dentine? yes, most assuredly, we would be very unwilling to be without it. The only unpleasant thing about it is, that there is some of our brethren who can not avail themselves of it; it will not dance when they whistle. Well gentlemen you don't whistle right.

Some in the profession regard this agent as very valuable, while others esteem it as totally worthless, for sensitive dentine at least. This would seem a strange thing were it not that there are many parallels.

### PERSONAL.

The fact stated on another page that the Editor of the REGISTER has accepted a position as teacher in the Dental Department of Michigan University has lead to some inquiry as to how it will affect his other engagements. In reply to such inquiries we will say that it will make no interruption in the course of instruction on Operative Dentistry in the Ohio Dental College, this will be given just as usual except that a little change in the order of the lectures will be necessary. It will not in the least interfere with the work upon the REGISTER, except perhaps improve it a little we hope.

Other matters will receive attention about as heretofore. A season of freedom for a few days each month, from the pressing cares of business will be very agreeable.

### OHIO DENTAL COLLEGE.

The clinic rooms of this institution will be open for the reception of students on the 1st of Sept. next, when the advantages of this department will be open to all who may come; they will be under the direction of suitable instructors, and are required to engage themselves upon whatever may be placed before them. Let all come who can.

